# Pruritic Vesicular Eruption on the Lower Legs in a Diabetic Female

**DOI:** 10.1155/2013/641416

**Published:** 2013-10-01

**Authors:** Hassan Riad, Hamda Al Ansari, Khaled Mansour, Haya Al Mannai, Hussein Al Sada, Samya Abu Shaikha, Sharifa Al Dosari

**Affiliations:** Dermatology Department, HMC, Doha, Qatar

## Abstract

A 50-year-old diabetic female presented with highly pruritic vesicles and excoriated lesions over the anterior aspect of both lower legs. The lesions were recurrent over the last two years. She received a lot of medications with partial response. Hb A1c was 10.8% (normal up to 7%). CBC showed microcytic, hypochromic anemia. Serum zinc, folate, IgE, TSH and T4 were all within normal ranges. Biopsy showed epidermal separation secondary to keratinocyte necrosis and minimal monocytic, perivascular infiltrate. Direct immunofluorescence was negative for intraepidermal and subepidremal deposition of immunoglobulin. The dermis was positive for mucin deposition stainable by both PAS and Alcian blue while it was negative for Congo red and APC immunoperoxidase staining for amyloid material. In conclusion, the case was diagnosed as bullosis diabeticorum by distinctive clinical and pathological features and after exclusion of other possible differentials. Pruritus was partially controlled by topical potent steroid and the case was resolved spontaneously after eight months.

## 1. Introduction

Bullosis diabeticorum is a distinct, spontaneous, non-inflammatory, blistering condition of distal and acral skin, unique to patients with diabetes mellitus. Krane first reported this condition in 1930; Cantwell and Martz are credited with naming the condition in 1967 [1]. It also is termed bullous disease of diabetes and diabetic bullae [2].

The etiology of bullosis diabeticorum is unknown. The role of trauma has been speculated; however, this alone does not explain spontaneous development of multiple lesions at different locations. Bullosis diabeticorum is a rare complication of long-standing diabetes mellitus. Patients with this disorder manifest a sudden onset of intraepidermal or subepidermal blisters, which are primarily confined to the extremities. However, it is not correlated with any specific sign of the disease except for its increased incidence with diabetic neuropathy [3–5]. 

Searching by title on PubMed, there were only 28 published results for bullosis diabeticorum, 2 results were for diabetic bullae, and no study was titled as bullous disease of diabetes. We believe that cases were underreported for many reasons: the benign course of the disease and lack of self-report by the patients. The condition occurs more frequently in middle age males with long-standing diabetes and neuropathy. Male-to-female ratio is 2 : 1, while the incidence among diabetics is 0.5% in the United States and 1% in India [6, 7], which constitutes a large number of patients. The disease is not yet studied in depth, and negative laboratory results exceeded positives. We hereby report a case that shared many features of the classic disease with minor differences. 

## 2. Case Report

**Figure 1:**
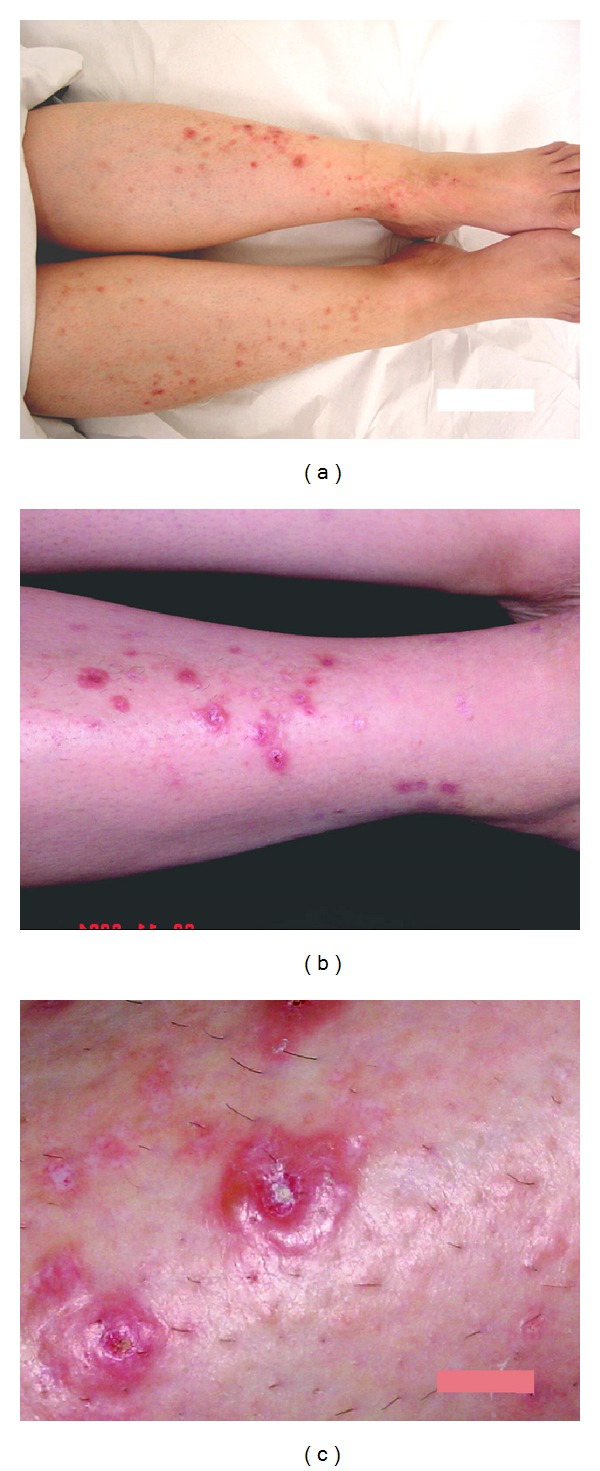
(a) The distribution of the lesions, bilateral and almost symmetrical, on both lower limbs. (b) Higher magnification showing the vesicular lesions lying on erythematous base and the fine white scars in healed lesions. (c) A close-up view of the vesicular lesions showing tense blistering with central crustations, on the right side of the photo a superficial white scar of healed lesion.

 A 50-year-old diabetic female presented with pruritic and recurrent skin lesions of two-year duration. She had uncontrolled type 2 diabetes for the last twenty years. The condition responded partially to systemic and local therapies. The skin lesions sized from 3 to 7 mm in diameter, distributed symmetrically over the anterior aspects of both legs, and extended to the dorsal aspect of both feet. They started as multiple, scattered, discrete, and tense vesicles surrounded by erythematous base. No history of hair removal or trauma. Some lesions were umbilicated with central crust, and others were excoriated as result of intense pruritus. Healing occurred with white fine depressed scars (Figure 1). 

**Figure 2:**
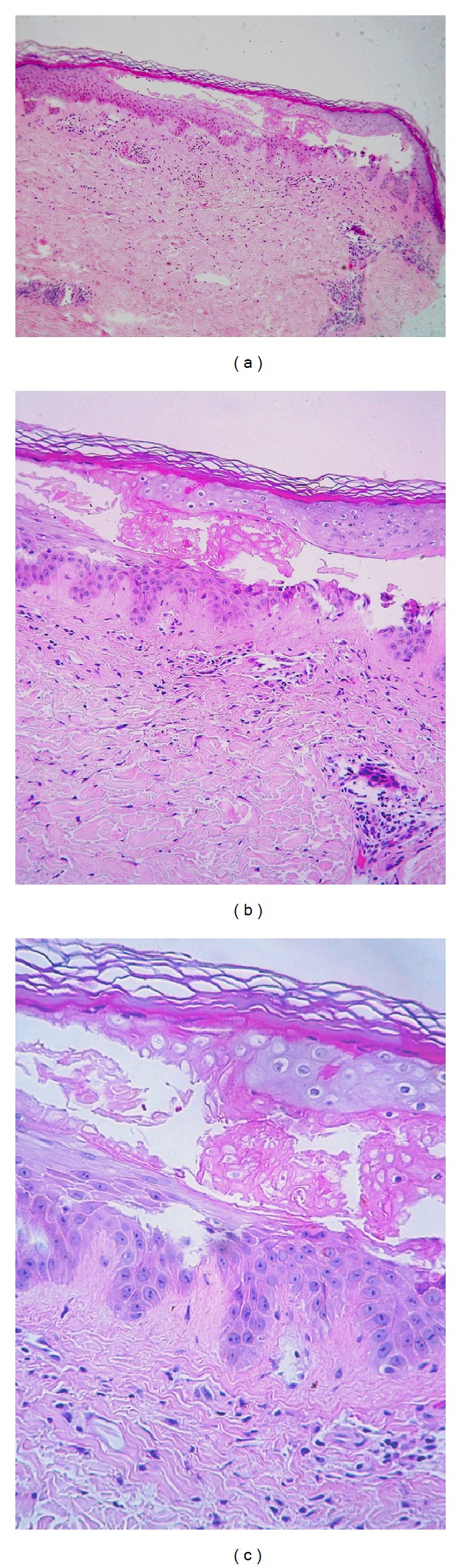
(a) H&E section, ×100 magnification showing an acute totally intraepidermal blister. At both angles of the blister, the separation is entirely intraepidermal. Stratum corneum is intact which attests the acute onset of the lesion, and no signs of regeneration can be seen. No evidence of common pathological changes that induce intraepidermal blister as acantholysis or ballooning degeneration. No inflammatory cells in or around the lesion. (b) H&E section, ×200 magnification showing epidermal separation at the level of the spinous layer. Necrotic individual keratinocytes can be seen intravesicular and at the roof of the blister (no acantholysis, no ballooning or multinuclear cells). (c) H&E section, ×400 magnification showed sparse perivascular infiltrate, and collagen bundles in the papillary dermis are separated by scanty mucin deposition.

Random blood sugar was 14.2 mmol/mL, and glycosylated hemoglobin Hb A1c was 10.8% (normal up to 7%). CBC showed microcytic hypochromic anemia. Serum zinc, serum folate, IgE, TSH, and T4 were all within normal ranges. Urine was negative for uroporphyrins. Biopsy showed acute, totally intraepidermal separation secondary to keratinocyte sudden death (both angles of the blister the separation are entirely intraepidermal). Separation occurred at the level of the spinous layer. Stratum corneum was intact which proves the acute onset of the lesion. Necrotic keratinocytes can be seen inside the vesicle and at its roof. No other pathological changes as acantholysis, ballooning or multinuclear cells, signs of healing, or regeneration were seen. Mild monocytic perivascular infiltration was found in upper dermis. No inflammatory cells in or around the lesion (Figure 2). 

The dermis showed deposition of faint mucin positive with both PAS and Alcian blue while it showed negative staining to Congo red and APC immuno-peroxidase staining for amyloid material. Direct immunofluorescence was negative for intraepidermal and subepidermal deposition of immunoglobulin. Diabetic angiopathic changes were lacking in the examined specimen. Smears and cultures from lesions were negative for both bacteria and fungi. Pruritus was partially controlled by topical potent steroid. Lesions healed with mild scar formation. The case was resolved spontaneously after eight months.

## 3. Discussion

The hallmark of the diagnosis was based on the clinical and pathological correlation. Other differential diagnoses considered were bullous pemphigoid, friction or physical blisters, bullous fixed drug eruption, bullous SLE, epidermolysis bullosa acquisita, localized amyloidosis, porphyria cutanea tarda, and dermatitis artefacta. Intraepidermal separation may also occur in blisters of metabolic origin as in pellagra, acrodermatitis enteropathica, and necrolytic migratory erythema. They are characterized by necrotic keratinocytes with mild dermal infiltrate and intact basal cells. Routine pathological examination of diabetic bullae may show similar features as intraepidermal or subepidermal bulla, keratinocyte necrosis, with little or no inflammatory infiltrate in the absence of any other possible clue of the disease.

 Comparing our case with other cases (see Table 1), our case presented with pruritus which is common in diabetics before developing sensory neuropathy. Most cases in the literature were cases of long-standing diabetes with neuropathy. The sensation in our case was still intact. In concordance with our case, blisters in some studies were intraepidermal in location [6, 7, 10, 11] while in majority of cases, blisters were subepidermal [8, 9]. Bullosis diabeticorum develops usually without inflammatory base, though in our case, lesions were mildly inflamed even in the untouched or unscratched new lesions.

Despite the intraepidermal level of the blister healing occurred with thin, atrophic scars (Figure 1) and this may be explained by the poor healing ability in some cases of diabetes. Also, the anatomical site can explain easy scar formation, because dermal fibrocytes of the skin of the anterior shaft of the tibia respond to trauma with excessive deposition of collagen and ground substance more than other areas in the lower limb. Deposition of mucin and over the shin of the tibia in diabetics is not uncommon. Other metabolic conditions were excluded by special stains and specific tests. Negative immunofluorescence completed the distinctive picture of the disease.

There is no effective therapy for this condition; nevertheless, control of diabetes is mandatory. Symptomatic relief occurs from time to time, and topical potent steroid showed some control over pruritus, but the patient is not satisfied because she did not get a radical cure for her problem.

In conclusion, we presented here a case of bullosis diabeticorum which is a rare skin manifestation of diabetes mellitus; it is a specific cutaneous marker and may present with intense pruritus and intraepidermal separation. Healing may occur with scar formation. There is no apparent cause for this condition; however, trauma and lack of control of diabetes are recognized predisposing factors.

## Figures and Tables

**Table 1:** Showing the different manifestations of bullosis diabeticorum described in the literature [8, 9] in comparison with our case.

Common findings in published cases of bullosis diabeticorum	Our case
Long-standing diabetes mellitus	Yes
Blisters occur spontaneously and abruptly, usually without known antecedent trauma	Yes
Lesions tend to be asymptomatic, despite mild discomfort or burning	Lesions were pruritic
Associated with neuropathy	No neuropathy
Blisters heal spontaneously within 2–6 weeks of onset	Lesion took more time to heal and were more persistent
Tense blisters	Yes
Evolve on nonerythematous or normal appearing skin	Lesions erupted on erythematous base
Mild or no scarring	Yes, depressed thin scars
Blisters typically occur on the feet or lower legs	Yes
Blisters tend to be large, size ranging from few millimetres to several centimetres	3–7 mm with no tendency to coalesce
Negative immunofluorescence: no primary immunologic abnormality exists	Yes
The blister plane may appear in a subcorneal, intraepidermal, or subepidermal location	Yes, blisters were intrepidermal
Presence of degenerative and necrotic keratinocytic	Yes
Absence of urinary uroporphyrins	Yes
Recurrence of condition is common	Yes
More in males	Our case was a female
